# Biomechanical comparison of anterior lumbar interbody fusion: stand-alone interbody cage versus interbody cage with pedicle screw fixation - a finite element analysis

**DOI:** 10.1186/1471-2474-14-220

**Published:** 2013-07-26

**Authors:** Kyung-Chul Choi, Kyeong-Sik Ryu, Sang-Ho Lee, Yeong Hyeon Kim, Sung Jae Lee, Chun-Kun Park

**Affiliations:** 1 Department of Neurosurgery, Wooridul Spine Hospital, Seoul, South Korea; 2 Department of Neurosurgery, Seoul St. Mary Hospital, The Catholic University, 505 Banpo-dong Secho-gu, Seoul 137-040, South Korea; 3 Department of Biomedical engineering, Inje University, Gimhae, South Korea

**Keywords:** ALIF, Stand-alone cage, Pedicle screw fixation, Finite element analysis

## Abstract

**Background:**

Anterior lumbar interbody fusion (ALIF) followed by pedicle screw fixation (PSF) is used to restore the height of the intervertebral disc and provide stability. Recently, stand-alone interbody cage with anterior fixation has been introduced, which eliminates the need for posterior surgery. We compared the biomechanics of the stand-alone interbody cage to that of the interbody cage with additional PSF in ALIF.

**Methods:**

A three-dimensional, non-linear finite element model (FEM) of the L2-5 segment was modified to simulate ALIF in L3-4. The models were tested under the following conditions: (1) intact spine, (2) destabilized spine, (3) with the interbody cage alone (type 1), (4) with the stand-alone cage with anterior fixation (SynFix-LR®; type 2), and (5) with type 1 in addition to PSF (type 3). Range of motion (ROM) and the stiffness of the operated level, ROM of the adjacent segments, load sharing distribution, facet load, and vertebral body stress were quantified with external loading.

**Results:**

The implanted models had decreased ROM and increased stiffness compared to those of the destabilized spine. The type 2 had differences in ROM limitation of 8%, 10%, 4%, and 6% in flexion, extension, axial rotation, and lateral bending, respectively, compared to those of type 3. Type 2 had decreased ROM of the upper and lower adjacent segments by 3-11% and 3-6%, respectively, compared to those of type 3. The greatest reduction in facet load at the operated level was observed in type 3 (71%), followed by type 2 (31%) and type 1 (23%). An increase in facet load at the adjacent level was highest in type 3, followed by type 2 and type 1. The distribution of load sharing in type 2 (anterior:posterior, 95:5) was similar to that of the intact spine (89:11), while type 3 migrated posterior (75:25) to the normal. Type 2 reduced about 15% of the stress on the lower vertebral endplate compared to that in type 1. The stress of type 2 increased two-fold compared to the stress of type 3, especially in extension.

**Conclusions:**

The stand-alone interbody cage can provide sufficient stability, reduce stress in adjacent levels, and share the loading distribution in a manner similar to an intact spine.

## Background

Anterior lumbar interbody fusion (ALIF) using a stand-alone interbody cage has been actively performed since the beginning of the 1990s [1,2]. However, when examining the research that has been performed to date, the results showed that ALIF has provided less than desirable stability in unstable spinal segments [3]. Presently, it is common to clinically perform an additional posterior fixation to recover the stability of spinal segments, as well as to enhance the fusion. If sufficient stability can be provided in a single surgery, the problems that can arise from extensive anterior and posterior approaches can be reduced [4]. This single surgical procedure could reduce the postoperative pain and number of days of hospitalization, and could lead to a quick return to one’s daily routine. Recently, a stand-alone interbody cage (SynFix-LR®; Synthes Gmbh, Oberdorf, Switzerland) with reinforced fixation from forward screws and a metal plate has been introduced; however, there is insufficient systematic biomechanical research to confirm the stability of an interbody fusion and increased fusion. SynFix-LR® consists of a polyetheretherketone (PEEK) body with an additional connected metal plate and a diverging locking metal screw; this design is meant to provide strong support and fixation to an unstable spine so that an additional posterior fusion would not be required.

Using finite element model (FEM) analysis, this study analyzed the range of motion (ROM) and facet joint load of the operated and adjacent segments, as well as changes in the anterior and posterior load in a normal model and in anterior lumbar interbody fixation performed using various methods. Through this analysis, the biomechanical effects of an ALIF for the stand-alone interbody cage were revealed.

## Methods

### Finite element model (FEM) of a normal lumbar spine

To develop a 3-dimensional (3D) FEM of the lumbar spine, computerized tomography was performed in 1 mm intervals on the lumbar spine (L2-L5) of an adult with no lesions. The FEM consisted of the vertebral body (cancellous bone and cortical bone), spinous process, intervertebral disc, and 7 types of ligaments (anterior longitudinal ligament, posterior longitudinal ligament, ligamentum flavum, capsular ligament, intertransverse ligament, interspinous ligament, and supraspinous ligament). Elastic behavior of the annulus fibers was taken from information provided by Smit et al. [5] who combined material values from Goel et al. [6] and Shirazi et al. [7]. The nonlinear behavior of the ligaments was incorporated by defining different material properties at different strains. Each location of the ligament was established according to the reference and anatomy data. Based on the research by Goel et al. [6], the gap between the facet joints was set to 0.5 mm, and the contact direction was set perpendicular to the articular surface. Material properties were selected from various sources in the literature (Table 1) [5-8]. This research used PATRAN (MSC Software Corp., LA, USA), a pre-post processing program, and ABAQUS (version 6.5, ABAQUS Inc., Providence, RI, USA), a general purpose finite element program.

**Table 1:** Material properties used in finite element model of lumbar spine

**Bony structures**	**Young’s modulus**		**Poisson’s**	**Reference**
	**E (MPa)**		**ratio**	
Cortical bone	12,000		0.3	Shirazi et al. [7]
Cancellous bone	100		0.2	
Posterior element	3,500		0.25	
End plate	25		0.25	Sharma et al. [8]
Annulus ground	4.2		0.45	
Nucleus pulposus	1.0		0.499 (incompressible)	Goel et al. [6]
**Annulus fibers**	**Young’s modulus**		**Cross-sectional**	**Reference**
	**E (MPa)**		**Area (mm2)**	
Layer 1/2	550		0.50	Combined from Shirazi et al. [7] and Smit et al. [5]
Layer 3/4	495		0.39	
Layer 5/6	413		0.31	
Layer 7/8	358		0.24	
**Ligaments**	**Young’s modulus**		**Cross-sectional**	**Reference**
	**E (MPa)**		**Area (mm2)**	
ALL	7.8 (<12%)	20 (>12%)	63.7	Adapted from Goel et al. [6]
PLL	10 (<11%)	20 (>11%)	20	
LF	15 (<6.2%)	19 (>6.2%)	40	
CL	7.5 (<25%)	33 (>25%)	30	
ITL	10 (<18%)	59 (>18%)	1.8	
ISL	10 (<14%)	12 (>14%)	40	
SSL	8 (<20%)	15 (>20%)	30	

Abbreviations: *All* anterior longitudinal ligament, *PLL* posterior longitudinal ligament, *LF* ligamentum flavum, *CL* capsular ligament, *ITL* intertansverse ligament, *ISL* interspinous ligament, *SSL* supraspinous ligament.

### Realization of the implanted model

**Figure 1:**
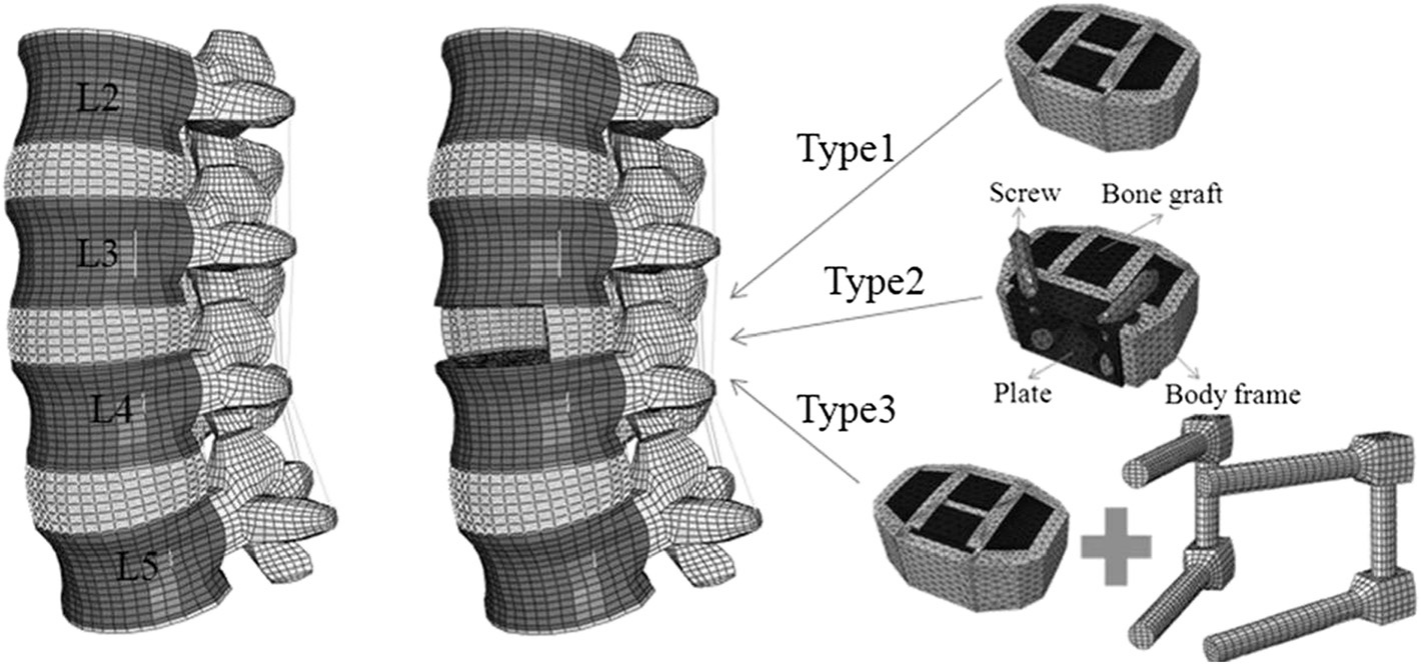
Three-dimensional finite element model of (A) a normal spine model (L2–5), (B) a destabilized model, and (C) post-operated models: type 1 (SynCage-LR®), type 2 (SynFix-LR®), and type 3 (SynCage-LR® + pedicle screws).

Three types of spine fixation devices used in this study were analyzed using the FEM (Table 2). The following models were used (Figure 1): (1) intact spine; (2) destabilized spine (anterior discectomy, removal of the anterior longitudinal ligament, and removal of the anterior and lateral annulus fibrosis); (3) insertion of an interbody PEEK cage (SynCage-LR®, Mathys Medical Ltd, Bettlach, Switzerland; type 1) on model 2; (4) insertion of a stand-alone PEEK cage (SynFix-LR®, type 2) reinforced with an anterior metal plate (Ti6Al7Nb) and 4 screws (Ti6Al7Nb); and (5) SynCage-LR® plus a posterior pedicle screw (TSRH, Medtronic Sofamor Danek, Memphis, TN; Ti6A14V, Φ = 5.5 mm; type 3). The SynFix-LR® and SynCage-LR® were constructed to a depth of 30 mm, width of 38 mm, and height of 13.5 mm, taking into consideration the size of the intervertebral disc in the 3D lumbar FEM used in this research. In this study, a higher friction coefficient of 0.8 was applied to the interface of the bone and cage after the surgical procedure in the implanted models, and it was hypothesized that bone adhesions develop between the bone and screw, which prevents the implant from moving [9]. Therefore, a tie contact condition was applied with the hypothesis that the SynFix-LR® and pedicle screws in the FEM are completely confined to the vertebral body.

**Table 2:** Material properties used in finite element model of spinal fixation systems

**Material**	**Young’s modulus**	**Poisson’s ratio**
PEEK	3.6 GPa	0.3
Ti6A17Nb	114 GPa	0.3
Ti6A14V	114 GPa	0.3

### Load conditions and boundary conditions

A multi-segment spinal model from L2 to L5 was used to compare and analyze the ROM of the operated and adjacent segments. All nodal points of the lower end plate of the lowest segment were confined, while the upper end plate of the highest segment was subjected to a pure moment of 10 Nm of flexion/extension/axial rotation and a pure moment of 5 Nm of lateral bending. A compressive force of 400 N was added to the validated intact lumbar spinal model in the follower load path direction as suggested by Patwardhan et al. [10].

### ROM and stiffness of the models

Changes in the ROM in the operated and adjacent segments (L2–3 and L4–5) with regard to exterior load were measured before and after implanting the spinal fixation device. The hybrid test protocol [11,12] was used to assess ROM at the operated and adjacent levels. The hybrid protocol has the following 2 steps: (1) application of a pure moment to the models and determination of total ROM and (2) application of the pure moment to the postoperative model in a displacement control mode until its ROM equals that of the intact models. At this time, pure moment applied to the postoperative model was defined as the resulting moment. The resulting moment was measured to confirm the effects of each variable element on stiffness of the spine. Subsequently the changes in ROM characteristics were investigated.

### Load sharing distribution

To investigate the effect on the anterior-posterior load-sharing ratio of the vertebral body, the intact spine and each implanted model were placed under 400 N of compressed loading, and the resulting values were compared and analyzed. We measured forces at the node on the x, y, and z axes of the anterior and posterior elements. The magnitude of the forces was calculated by adding each measured nodal force and confirmed load-sharing ratio. We did not consider the influence of the ligaments.

## Results

### Verification of the FEM

**Figure 2:**
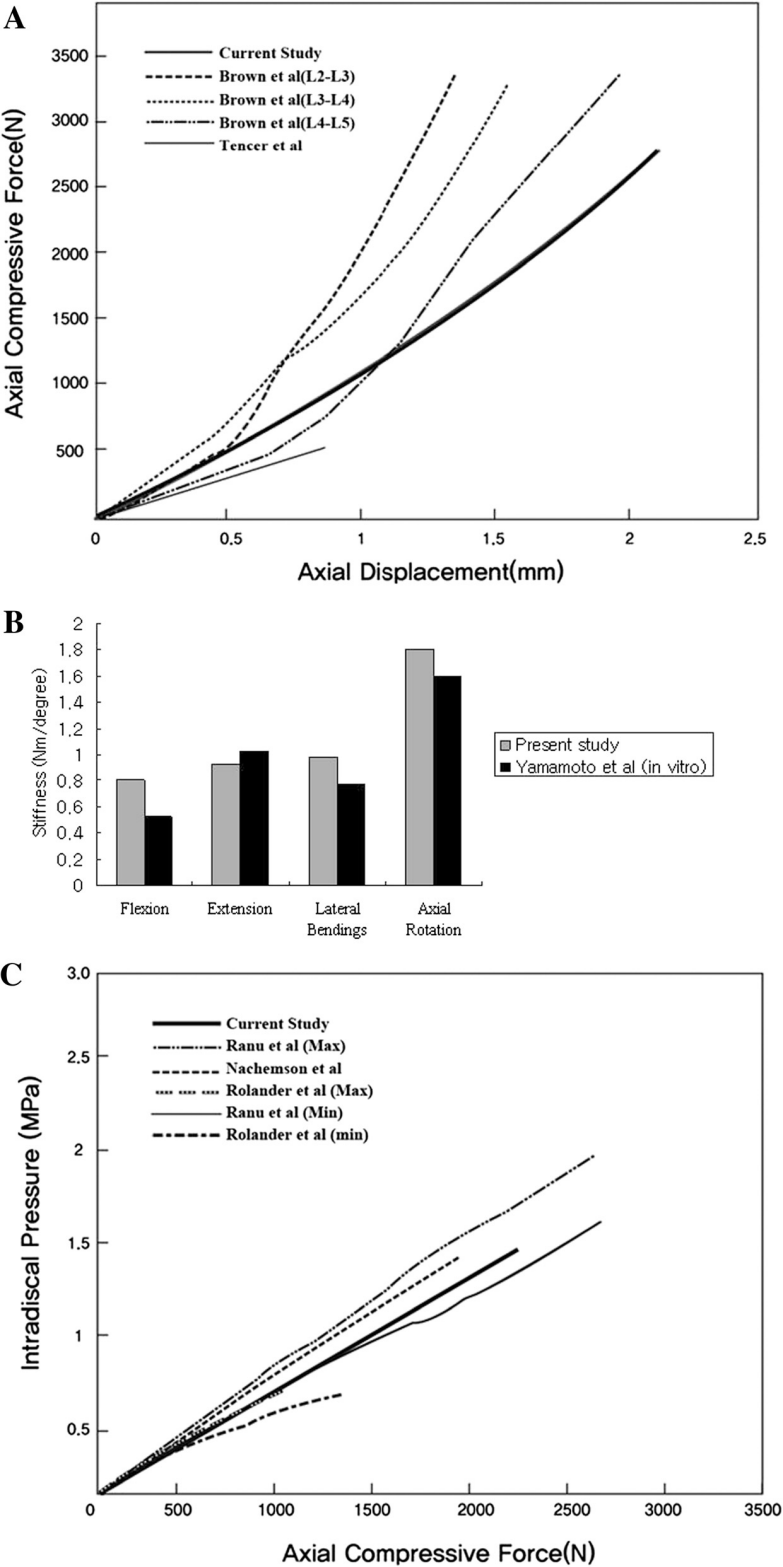
Intact model validation based on cadaveric study: stiffness under axial compressive load (A), various loading modes (B), and intradiscal behavior under axial compressive load (C).

**Figure 3:**
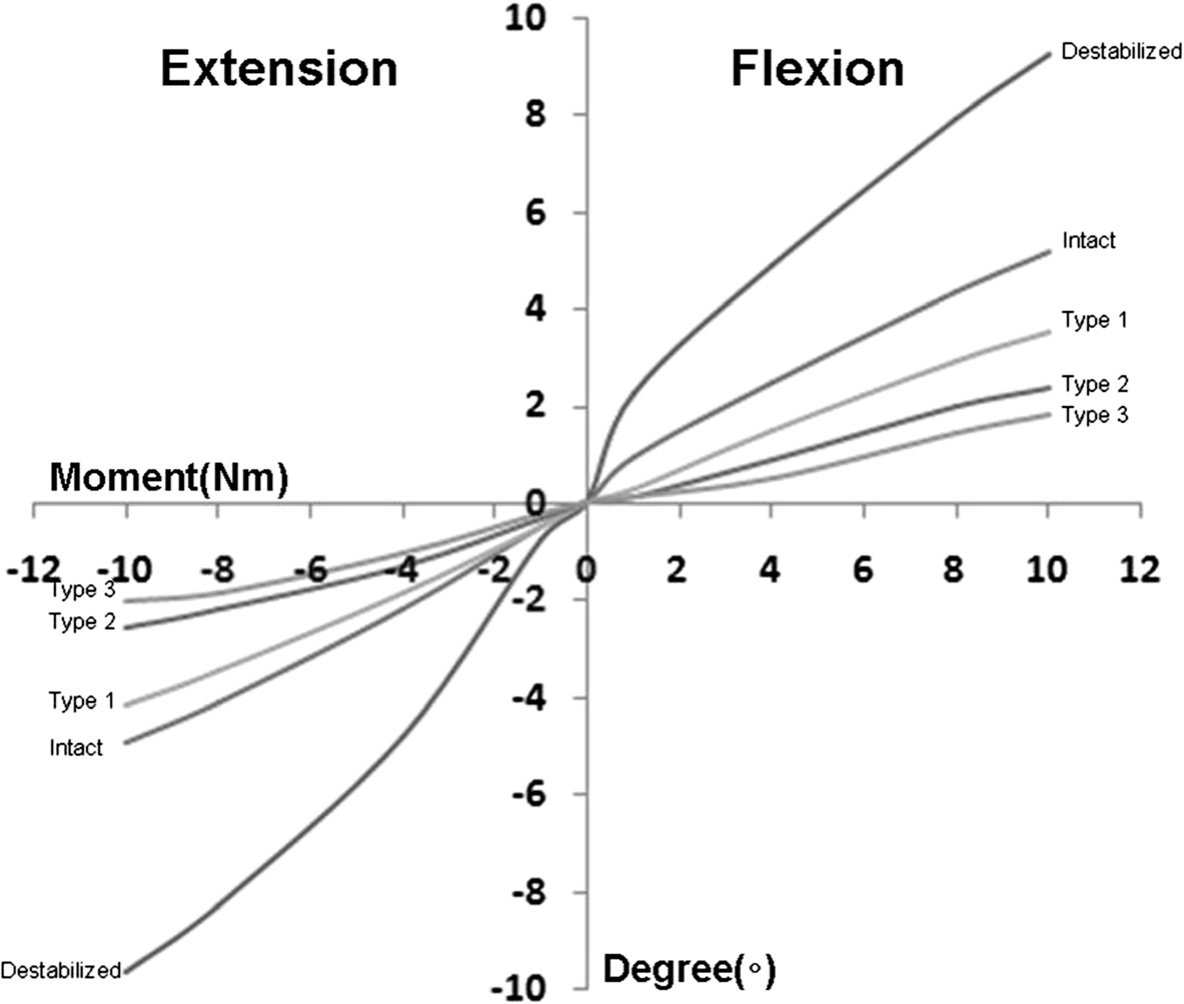
Moment-rotation curve in flexion/extension with 5 models (destabilized spine, intact spine, type 1, type 2, and type3) at an operated segment (L3–4) in 10 Nm flexion/extension superimposed a follower load of 400 N.

The lumbar FEM in this study was verified by referencing the spinal motion analysis performed by Yamamoto et al. [13]. The resulting values of the finite element interpretation were similar to the experimental results in the literature [13-15]; therefore, the FEM used in this research proved to be valid (Figure 2A, 2B). The calculated intradiscal pressure value was linear, and it was confirmed to increase proportional to the compression loading. It was nearly identical to the experimental results reported in the existing literature [16-18] and as a result, is highly credible. Thus, the values resulting from the FEM are credible (Figure 2C). The FE study about the lumbar spine adopted nonlinear load–displacement relationship (Figure 3).

### ROM and stiffness of the models

**Figure 4:**
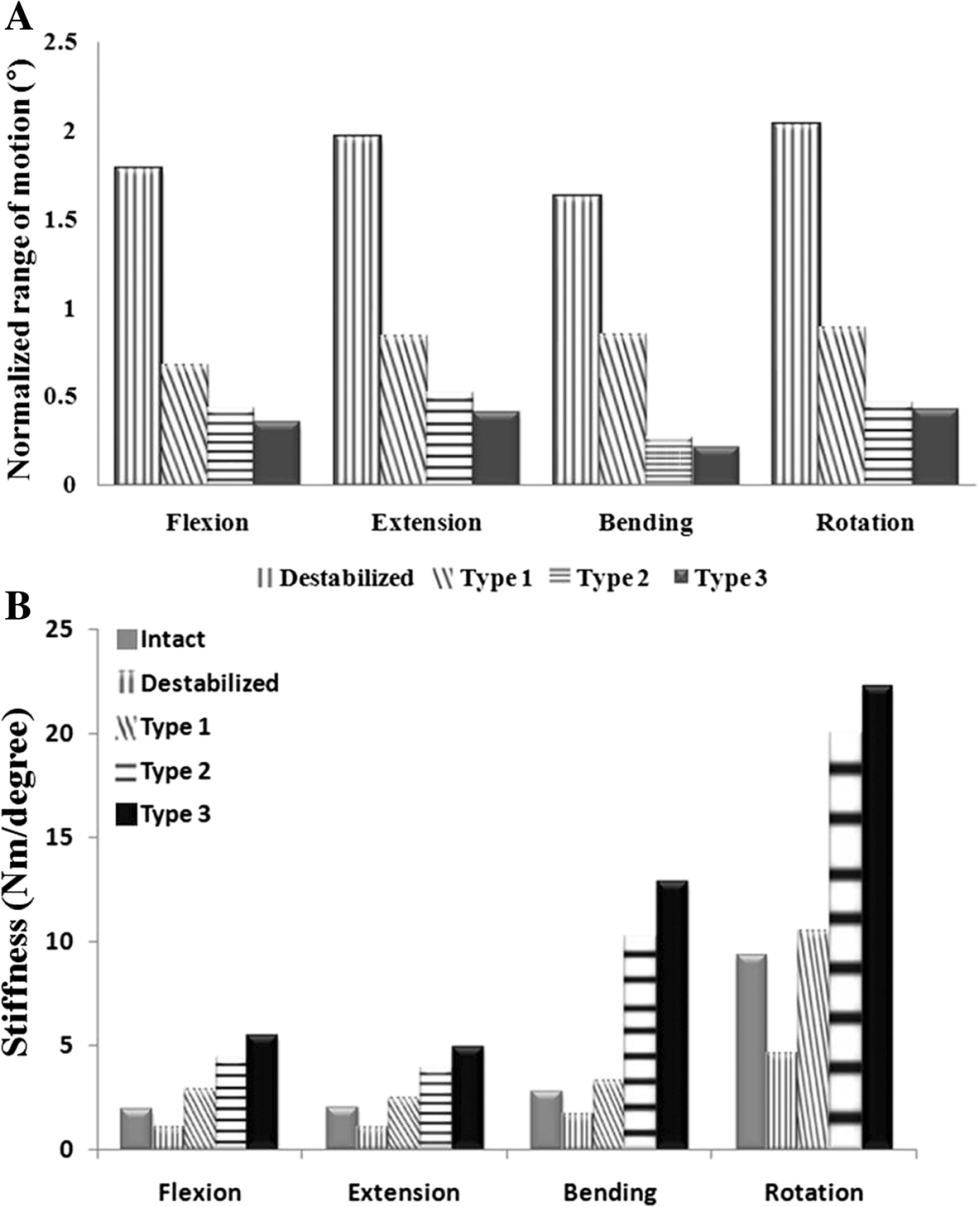
ROM (A) and stiffness (B) among various surgical models at the operated level under flexion, extension, lateral bending, and axial rotation.

The stiffness (Nm/degree) of the operated segment was expressed using the measured ROM of the operated segment (Figure 4). In all the implanted models, the ROM decreased in the operated segments. The greatest reduction of ROM occurred in type 3, where the ROM was limited up to 79% compared with normal ROM. Excluding flexion, the ROM for the operated segment in type 1 had less than a 15% limitation in ROM, which was the smallest change among all the implanted models. In addition, a minimum ROM increase of 60% (maximum, 103%) was observed in the destabilized model. Type 2 had a ROM limitation of about 73% compared with the normal when lateral bending was applied. Additionally, type 2 had differences in ROM limitation of 8%, 10%, 4%, and 6% in flexion, extension, axial rotation, and lateral bending, respectively, compared to those in type 3. In the operated segments, a difference in stiffness was observed in decreasing order for type 3, type 2, and type 1, and the difference between the type 2 and type 3 was not large.

### Adjacent segment motion

**Figure 5:**
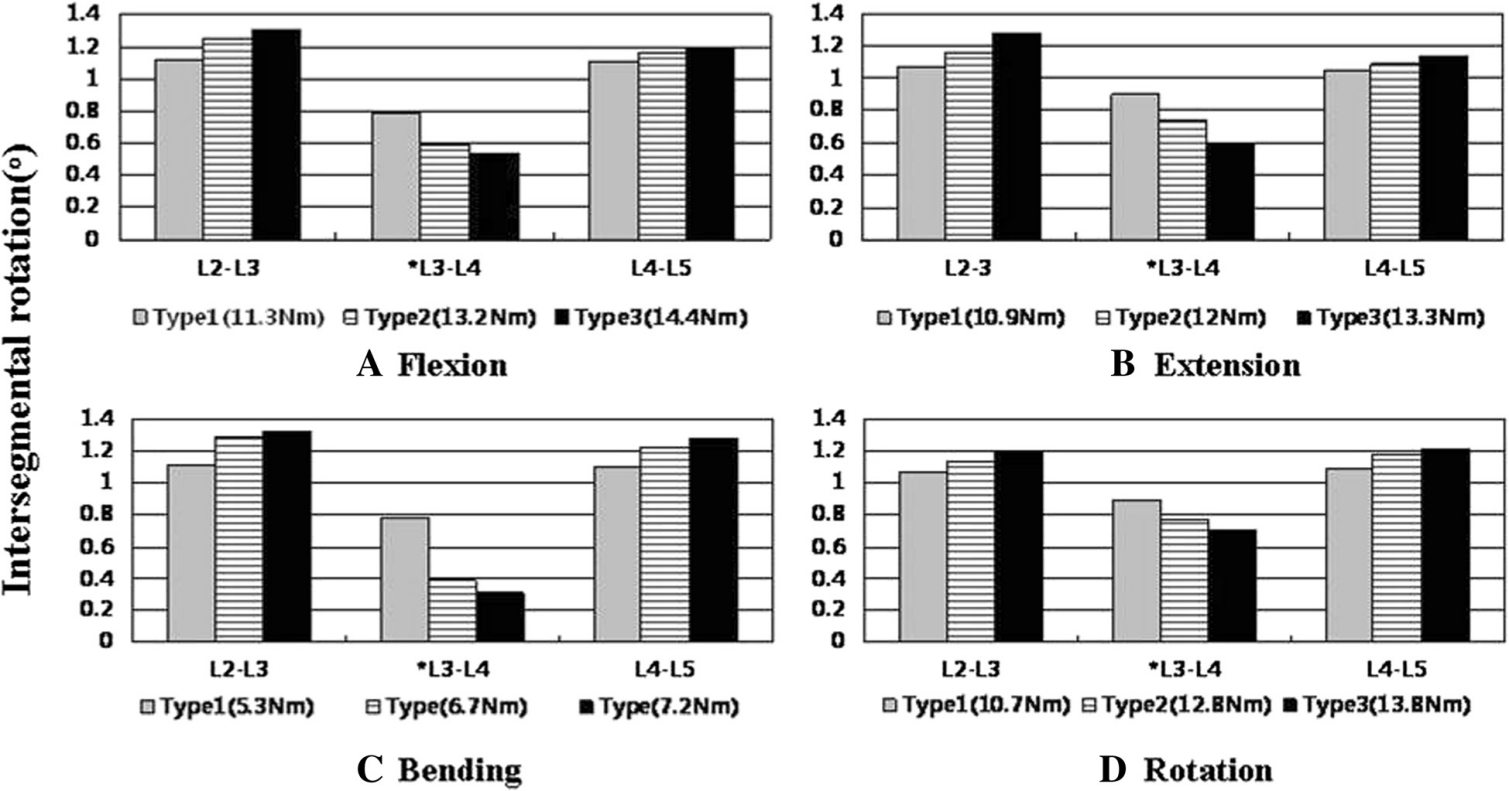
**Normalized intersegmental rotation angle of types 1, 2, and 3 in flexion (A), extension (B), bending (C), and axial rotation (D) using a hybrid multidirectional test [**[11]**].**

Type 1 had nearly half the amount of motion in the adjacent segments compared to that in type 3 under all given conditions, and had the least amount of increase in the adjacent segments among all the implanted models. Type 2 showed a relatively small increase in the ROM of the adjacent segments in all given conditions compared to those in type 3; among these, the difference in the upper segment motion was approximately 11% when extension was applied. Additionally, type 2 showed a decrease in ROM of the upper and lower adjacent segments by 3–11% and 3–6%, respectively, compared to those of type 3 (Figure 5).

### Load sharing distribution and facet load

The normal shared load was 89% anteriorly and 11% posteriorly. The ratios of anterior to posterior load sharing in types 1, 2, and 3 were 94:6, 95:5, and 75:25, respectively. The facet load in the implanted models decreased the most in type 3 (71%), followed by type 2 (31%) and type 1 (23%). The facet loads in the adjacent segments (L2–3) had similar pressures in type 1 and type 2, while type 3 had higher pressures than the other types.

### Peak von Mises stress in the lower vertebral body

**Figure 6:**
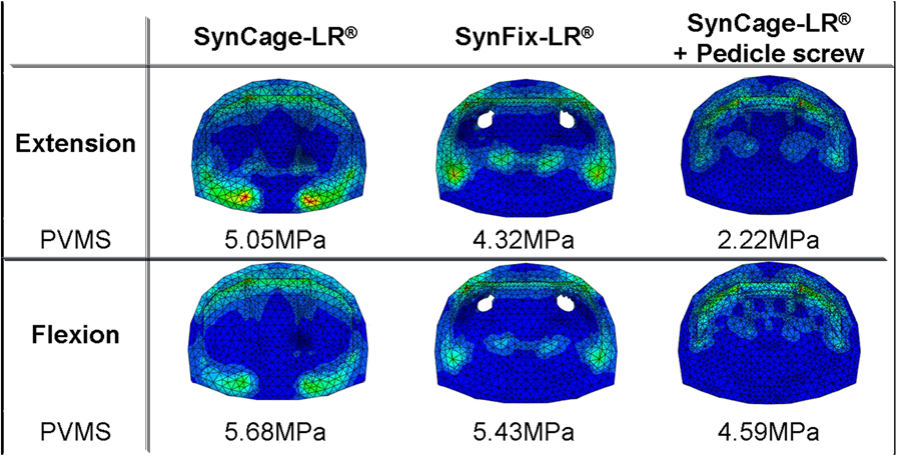
Contour plots of peak von Mises stress (PVMS) of the lower vertebral body (L4) when tested with flexion load of 10 Nm (A) and extension load of 10 Nm (B) after applying a compressive follower load (400 N).

Peak von Mises stress (PVMS) of the superior surface of L4 which is a contact space from the flexion (10 Nm) and extension (10 Nm) after applying a compressive follower load (400 N) on the model are shown in Figure 6. Types 1, 2, and 3 had a PVMS of 5.68, 5.43, and 4.59 MPa in flexion, respectively. Additionally, types 1, 2, and 3 had a PVMS of 5.05, 4.32, and 2.22 MPa in extension, respectively.

## Discussion

Lumbar interbody fusion has an exceptionally high fusion rate, and correction and restoration of the spine is relatively simple compared to that in posterolateral fusion [19,20]. An ALIF with a cage is widely used to maintain the height of the intervertebral discs and to restore the stability of the spinal segments. Generally, posterior pedicle screw fixation is used in parallel to heighten the stability of the operated segment and fusion rate in ALIF [21]. However, there are several potential issues, including biomechanical changes from the anterior-posterior fusion, surgical risks and complications, increased cost of the surgery, and increased operative time due to the fixation of an additional PSF [1,22]. Additional posterior fixation after cage implantation provided superior biomechanical stability but increased the risk of neurological damage or damage to the muscle and ligament surrounding the spine from the additional posterior surgery. Therefore, questions have been raised as to whether this anterior-posterior fixation achieves better clinical results [4]. In the case of anterior fusion using stand-alone interbody cage, in several studies it has shown stability and resistance to flexion or lateral bending but was vulnerable to applied extension or axial rotation [4]. To overcome these problems, there have been studies in which an anterolateral plate is attached to the cage [23] or screws are cross-fixed between the femoral ring allograft and cancellous bone [24]. When the results of research using anterolateral or lateral metal plates and screws after cage implantation were examined, a vastly improved stability was noted. In a study using cadavers that compared implanting a femoral ring allograft between the vertebral body to cross-fixing screws attached to the femoral ring allograft to the upper and lower vertebral body, the experimental group with the screws holding the femoral ring had increased stiffness under all given loads compared to that in the group with only the femoral ring allograft during extension (52.9% vs. 16.9%) and axial rotation (40.2% vs. 18.3%) [24]. Using additional anterior fixation of the anterior cage, these studies were able to heighten the stability by supplementing the vulnerability regarding extension from the removal of the anterior longitudinal ligament, which had been the most vulnerable area of the stand-alone cage. In another study using cadaver segments between L5 and S1, a model using the anterior cage fixed with 3 screws and a triangular shaped metal plate, and a model using posterior fixation with pedicle screws were compared. Both models showed similar limitations in movement and stiffness in the operated segment. However, the anterior plate fixation model exhibited vulnerable stability to lateral bending compared to that exhibited by the model with the pedicle screws; this difference in the experimental results was presumed to arise from the difference in the number of fixed points [1]. In our study, the SynFix-LR® (in which the anterior fixation provided superior stability compared to that provided by just using the vertebral interbody cage [SynCage-LR®]), had a similar stiffness in the operated segment compared to that in the model with the pedicle screws, and a difference in limitation in ROM of less than 10%. When other research results regarding SynFix-LR® were examined, it was found that the SynFix-LR® had better stability in lateral bending, extension, and axial rotation when compared to those for a semicircle wedge-shaped stand-alone cage equipped with screws facing the center of the vertebral body (STALIF; Surgicraft Ltd, Redditch, UK). These differences in the results are presumed to be due to the variation in the direction of the screws (divergence vs. convergence), the characteristics of the screws (locking screw), and the cage shape [25]. Cain et al. [26] used a cadaver to study the biomechanics of SynFix-LR® similar to the experimental method used by the authors in this study. When comparing the results of the experimental group with the SynFix-LR® and additional PSF after cage implant, there were no significant differences when flexion, lateral bending, or extension were applied. However, SynFix-LR® was reported to exhibit a narrower elastic zone and stronger stiffness in the axial rotation. This is probably due to the convex cage body and strong adhesion with the vertebral body through the anterior fixation screws and metal plates with the 4 fixation points. This supplemented the stability during extension, axial rotation, and lateral bending, which had been a vulnerability of fixation procedures using stand-alone cages and anterior plates.

Degeneration of adjacent segments after fusion is a potential long-term complication. Limitation in motion occurs in the lumbar fusion region as a compensatory mechanism, and excessive movement occurs in the segments above and below the fusion region. Results from many studies showed that PSF increased the degeneration of the adjacent segments [27,28]. In posterior lumbar fusion, when the pedicle screws were removed after fusion, the movement of the adjacent segments for flexion, extension, axial rotation, and lateral bending increased relatively less than when the pedicle screws were retained, and the stress of the intervertebral disc of the adjacent segments also decreased more than 50%. When the pedicle screws were retained, a marked increase in the movement of the segments above the operated area was observed when flexion was applied [28]. This study confirmed that there were changes in the anterior-posterior load-sharing of the lumbar vertebrae after fusion [29,30]. When PSF was performed, the load was focused more posteriorly compared with a normal lumbar segment, and to compensate, the burden on the facet joints of the adjacent areas increased. On the other hand, the stand-alone cage model had a similar load sharing distribution to a normal lumbar segment, and had relatively less motion in the adjacent segments compared to that for the PSF. This was considered result from the stand-alone cage with the reinforced anterior fixation moving the load that had been applied to the posterior of the spine to the anterior, which relieved the heavy load that had been applied to the facet joints of the adjacent segments.

The SynFix-LR® showed an approximate decrease of 15% in applied stress to the lower endplate compared to that using only the SynCage-LR® while in flexion. However, when extension was applied, the additional PSF model received half the PVMS of the stand-alone cage. When pedicle screws were used, the stress on the vertebral body moved anteriorly, while when only the SynCage-LR® was used, the stress was focused on the posterior part of the vertebral body. Through anterior fixation, the stand-alone cage can reduce the stress focused on the posterior of the vertebral body, as well as disperse the stress from surrounding vertebral body at the same time.

The limitation of this study is that the material properties of this simulation, such as the non-linear behavior of the spinal ligaments, the viscoelasticity of the disc, and the grade of degenerative disc, were simplified and idealized based on the properties of a cadaver specimen. This is due to the differences in the geometry and material properties, where the intervertebral discs within the human body are extremely flexible structures; however, the finite element simulated the intervertebral disc as a solid element. Thus, the non-linear characteristics were difficult to reenact. In addition, the state of the bone, which is an important factor to consider in fusion procedures, was not considered. Finite element research is similar to studies that are conducted *in vitro*, and as such, muscle contraction around the spine could not be considered.

## Conclusion

In conclusion, the stand-alone cage with reinforced anterior fixation provided sufficient stability and stiffness necessary to carry out lumbar fusion and at the same time, reduced the excessive motion of the adjacent segments and the stress on the adjacent segment joints by exhibiting similar load sharing characteristics to a normal lumbar. Therefore, the stand-alone cage can supplement with the shortcomings of the anterior lumbar interbody cage with no fixation device and have the advantage of parallel use with PSF.

## Competing interests

The authors declare that they have no competing interests.

## Authors’ contributions

Ryu KS, Lee SH, Park CK provided advice on the study design. Choi KC carried out the whole studies, participated in the sequence alignment and wrote the article. And Choi KC was responsible for the data acquisition/analysis, interpretation. Lee SH provided advice on the data analysis. Ryu KS, Lee SH, Park CK contributed to the content of the article. Kim YH, Lee SJ involved in surgical treatment. All authors read and approved the final manuscript.

## Pre-publication history

The pre-publication history for this paper can be accessed here:

http://www.biomedcentral.com/1471-2474/14/220/prepub
